# Evidence for conservation and sustainable use in a fragment of the Atlantic forest in southeastern Brazil by a traditional human group

**DOI:** 10.1186/2193-1801-1-21

**Published:** 2012-09-28

**Authors:** Alexandre Gabriel Christo, Rejan R Guedes-Bruni, FelipedeAraújoPinto Sobrinho, Ary Gomes da Silva, Ariane Luna Peixoto

**Affiliations:** 1 Jardim Botânico do Rio de Janeiro (JBRJ), Rua Pacheco Leão, 915, CEP 22.460-030 Rio de Janeiro, RJ Brazil; 2 Pontifícia Universidade Católica do Rio de Janeiro (PUC-Rio), Ciências Biológicas, Rua Marquês de São Vicente, 225, Prédio Pe. Leonel Franca, 7 andar, CEP 22451-900 Rio de Janeiro, RJ Brazil; 3 Universidade Vila Velha-ES (UVV), Rua Comissário José Dantas de Melo, 21, CEP: 29.102-770 Vila Velha, ES Brazil

**Keywords:** Ethnobotany, Atlantic forest, Rural community, Use-value, Forest resource, Conservation

## Abstract

The use of forest resources by a rural community adjacent to a Biological Reserve was examined using quantitative methods based on the consensus of six local specialists. Plants with trunk diameters at 1.3 m above soil level (DBH) ≥ 5 cm were sampled in 0.5 ha of forest and their use-value (UV) were calculated and associated with their structural descriptors. A total of 129 species were identified, and 69 of them having known uses. The species with largest UV were: *Xylopia sericea*, *Lecythis lanceolata* and *Guarea macrophylla*. The results demonstrated that neither the degree of recognition of taxa by the local specialists nor their use-versatility depended on their abundance in nature. The results corroborate the hypothesis that richness of a plant family is a predictive character of its cultural importance and the community recognizes the value of conserving the forest remnants.

## Introduction

Conservation of biological diversity represents one of the greatest current challenges for the mankind, due to the high degree of anthropogenic disturbance of all natural ecosystems al over the world ([Lawrence 2010]).

In Brazil, the land occupation, the degradation of natural landscape and the social aspects related to this context, has in the Amazonian region the greatest visibility both nationally and internationally, due to its geographic dimension, biological diversity and challenges of various origins ([Mittermeier et al. 2005]). Considering the megadiversity in the country ([Gross et al. 2005]), this forest is only a part of a bigger problem. The severe process of anthropization and subsequently suppression of vegetation areas had drastically reduced the limits of important Brazilian biomes ([Castelletti et al. 2003]), but no suppression was so severe such as the one that happened to the Atlantic Forest, limiting its remains to no more than 6% of its original area ([Fundação SOS Mata Atlântica 2009]).

The Atlantic Forest, due to the fact that it encompasses the geographic region where the first Brazilian cities were established – and nowadays, about 60% of the Brazilian population ([Fundação SOS Mata Atlântica 2009]) – was interconnected to the different economic cycles in the country, from the exploration of *pau-brasil* (*Caesalpinia echinata* Lam.), at the time the country was discovered, up to the incentive for the development of technology to produce bio fuel in the 70’s ([Guedes-Bruni et al. 2000]). The agriculture fostered since the period of colonial Brazil (XVI – XVII centuries), allied to the mining activity in the southern country, had already damaged the forest landscape to a huge extent ([Dean 1996]), and endangered many plant and fauna species. Hence, the scientific studies concentrate predominantly in protected areas, reminiscent of a forest which was once pungent along the Brazilian Atlantic coast.

Ethnobotanical studies directed towards examining the relationships between humans and the forest as well as the natural populations of plant species and the impacts caused by use and management by human populations have been undertaken by numerous authors ([Piñedo-Vásquez et al. 1990], [Phillips and Gentry 1993a, b], [Phillips et al. 1994], [Chazdon and Coe 1999], [Calderon et al. 2000], [Galeano 2000], [Aguilar and Condit 2001], Torre-[Cuadros and Islebe 2003]), but they are still incipient for a megadiverse country such as Brazil ([Prance et al. 1987], [Cunha and Albuquerque 2006], [Soler-Alarcón and Peixoto 2008]), and contain valuable information that can assist the management of useful species and the *in situ* conservation of threatened species.

Various studies have shown local knowledge to be extremely important in environmental diagnoses, especially in terms of its historical comprehension and long term observations ([Donovan and Puri 2004], [Cundill et al. 2005], [Chalmers and Fabricius 2007]). The knowledge retained by human populations living near remnant forests, especially those near conservation areas, can help indicate options for sustainable use and *in situ* conservation of valuable resources based on the history of that community and knowledge that it transmits orally from generation to generation ([Albuquerque 1999]).

This knowledge, as well as the Atlantic Forest itself, is threatened with extinction with the death or loss of memory of the oldest citizens, and by the disinterest of the younger community members who are now living in a globalized world with its emphasis on urban values ([Christo et al. 2006]). Ethnobotanical studies are important for strengthening the cultural identity of rural communities while at the same time strengthening concepts and scientific methodologies that synergistically influence the use and conservation of natural systems as demanded by public environmental policies ([Albuquerque et al. 2009]).

The present study sought to: (1) evaluate the use and importance of the arboreal species in an Atlantic Forest fragment as indicated by local specialists of a rural community near the Poço das Antas Biological reserve, in the municipality of Silva Jardim, Rio of Janeiro, Brazil, and (2) identify associations between the importance of these resources and their community structural absolute descriptors, such as dominance, density, and frequency.

### Study area

The Gleba Aldeia Velha rural community (22^o^30’20”S and 42^o^16’30”W) is in the municipality of Silva Jardim, on the central coast of Rio of Janeiro State, about 120 km distant from the state capital, and neighboring the Poço das Antas Biological Reserve where the predominant vegetation is Dense Ombrophilous lowland Atlantic Forest ([Veloso et al. 1991]), in different states of maturity ([Lima et al. 2006]).

Some of the inhabitants of the study community formerly lived in what is now part of the Reserve and were resettled along its borders after its creation at the end of the 1970’s. Gleba Aldeia Velha is composed of approximately 60 families whose economic activities are based on small-scale agriculture, principally planting manioc (*Manihot esculenta* Crantz) and corn (*Zea mays* L.), as well as on animal husbandry, raising cattle for milk and meat ([Christo et al. 2006]). Occasionally, some individuals take a part in activities developed by NGOs, universities and research institutions in Reserve.

## Methods

Field work was carried out between March 2007 and March 2008, when we interviewed six local specialists (men that retain specialized knowledge concerning the local native arboreal species) using questionnaires during semi-structured interviews ([Alexiades and Sheldon 1996]) focusing on their personal life histories and their relationships with, and use of, forest resources. Local specialists were 38 up to 72 year-old and they were harvesters in their majority, but sometime in the past, some of them had also worked in that area as loggers. Their instruction level was never higher than the fundamental education.

The informants were not randomly selected, as seen in [Phillips and Gentry (1993a]) but were chosen on the basis of indications from the community itself, taking into account their significant knowledge about the forest elements ([Christo et al. 2006]). A second semi-structured interview was later held with the same local specialists to obtain information about the use (use-categories and the plant parts utilized) of the plant species harvested and the localities where they were collected.

In order to identify possible associations between species use and their natural abundance and diversity, 20 sample plots (10 x 25 m), totaling 0.5 ha, were laid out, and all individuals with DBH ≥ 5 cm were recorded and, three branches of each species were collected to make vouchers that were deposited in the colletion of the Herbarium Barbosa Rodrigues, at the Rio de Janeiro Botanical Garden.Forest structural parameters and the analysis are found in [Christo et al. (2009]). The nomenclatural system adopted for plant families and species scientific names was the one of the Angiosperm Phylogeny Group – APGIII ([Bremer et al. 2009]). Additional ethnobotanical data was collected by way of a third series of interviews with the local specialist during walks in the sample plots. During these walks the men identified the plants by name and indicated their uses and the plant parts used. These ethnobotanical data were used to calculate the UV of each species *s* for each informant *i* (UV_is_) as well as the use-value of each species *s* (UV_s_), as proposed by [Phillips and Gentry (1993a]).

To calculate the use-value of the plant families, the methodology suggested by [Phillips and Gentry (1993a]) and modified by [Galeano (2000]) was adopted, in which the UV for each species s belonging to family *F* are summed, without dividing by the total number of useful species per family. The uses identified by the local specialists were grouped into nine categories: foods, medicinal, construction, firewood, ornamental, ritualistic, technological (comprises crafts made with wood, such as toys, tool handles, furniture, and housewares) and toxic. These same categories have been used in other ethnobotanical studies that have analyzed arboreal vegetation ([Prance et al. 1987], [Piñedo-Vásquez et al. 1990], [Phillips and Gentry 1993a, b], [Phillips et al. 1994], [Galeano 2000], [Torre-Cuadros and Islebe 2003]).

Plants included within the category “firewood” are normally grouped within the category “technology” ([Phillips and Gentry 1993a]), but here we considered them as distinct categories, in light of the importance that firewood use has for the local community - as identified by [Christo et al. (2006]), and corroborating with the categories adopted by [Galeano (2000]) and [Cunha and Albuquerque (2006]).

Linear regression analysis was used to identify the association between the importance of each species for the local specialists in relation to its availability, comparing the use-value of each species with the structural descriptors of density and dominance, and with the richness of the plant families.

The identification of the botanical families that were sub-utilized or highly used, in relation to their species richness, was performed by processing the residuals obtained by the linear regression calculated with an independent variable (richness) and a dependent variable (the use-value of the family), following [Phillips and Gentry (1993a]) and [Galeano (2000]). Assuming α = 0.05, the families that have residual values either greater or lesser than 1.96 were identified as families that are either super- or sub-utilized respectively in relation to the richness values obtained in the structural analysis of the forest ([Phillips and Gentry 1993a]).

Samples of all botanical material were collected with the indication of the informant and identified using analytical keys, taxonomic reviews, comparisons with herbarium collections and with the help of taxonomists. The classification system adopted follows APG III (2009). All voucher material was deposited in the herbarium of the Rio de Janeiro Botanical Garden (RB). See [Christo et al. (2009]) for A. G. Christo’s collection numbers.

## Results and discussion

In the fragment sampled, a total of 734 individual plants were recorded, belonging to 129 species in 41 families (Table 1). Concerning the ethnobotanical results, nearly 53.49% of the species (69) and 78.20% (574) of the individuals were recognized by the local specialists as having some kind of utility.

**Table 1:** **Use-value and structural descriptors of the arboreal species considered useful by local specialists in ruralcommunity, Brazil, presented in decreasing absolute dominance order (Ab.Dom)**

Species	Ab.Dom	Ab.Freq.	Ab.Dens	UV
*Piptocarpha macropoda* (DC.) Baker	1.1184	24.0	35.0	1.50
*Tapirira guianensis* Aubl.	1.1021	24.0	45.0	2.17
*Aparisthmium cordatum* Baill.	1.0587	102.0	75.0	1.20
*Guapira opposita* (Vell.) Reitz	1.0543	86.0	80.0	1.00
*Xylopia sericea* A. St.-Hil.	0.9801	34.0	55.0	3.40
*Albizia polycephala* (Benth.) Killip	0.8578	32.0	25.0	2.17
Malpighiaceae 1	0.8163	34.0	35.0	1.40
*Guatteria xylopioides* R.E.Fr.	0.7319	38.0	50.0	1.80
*Malouetia arborea* (Vell.) Miers	0.6146	6.0	15.0	1.80
*Cabralea canjerana* (Vell.) Mart.	0.5618	40.0	65.0	2.40
*Hieronyma oblonga* (Tul.) Müll. Arg.	0.5278	22.0	35.0	1.83
*Pogonophora schomburgkiana* Miers	0.5096	22.0	15.0	1.67
*Siparuna brasiliensis* (Spreng.) A. DC.	0.4940	44.0	55.0	1.20
*Lacistema pubescens* Mart.	0.4860	68.0	60.0	1.00
*Annona cacans* Warm.	0.4635	4.0	10.0	0
*Nectandra oppositifolia* Nees & Mart.	0.4608	22.0	30.0	1.67
*Virola oleifera* (Schott) A.C. Sm.	0.4349	14.0	20.0	1.75
*Alchornea triplinervia* (Spreng.) Müll. Arg.	0.4245	6.0	10.0	2.60
*Jacaranda micrantha* Cham.	0.3940	10.0	20.0	2.60
*Simarouba amara* Aubl.	0.3844	10.0	25.0	1.50
*Myrcia anceps* O. Berg	0.3752	56.0	45.0	1.17
*Cupania racemosa* (Vell.) Radlk.	0.3171	16.0	35.0	1.20
*Rinorea guianensis* Aubl.	0.3144	30.0	35.0	1.60
*Ocotea divaricata* (Nees) Mez	0.2905	36.0	55.0	1.67
Asteraceae 1	0.2899	6.0	15.0	1.00
*Aniba firmula* (Nees & Mart.) Mez	0.2739	32.0	45.0	2.50
*Chrysophyllum lucentifolium* Cronquist	0.2568	2.0	5.0	0
*Bathysa mendoncaei* K. Schum.	0.2440	46.0	45.0	1.00
*Ocotea schottii* (Meisn.) Mez	0.2214	10.0	15.0	1.00
*Miconia cinnamomifolia* (DC.) Naudin	0.1954	8.0	15.0	1.60
*Eugenia oblata* Roxb.	0.1925	8.0	15.0	1.80
*Rollinia dolabripetala* (Raddi) R.E. Fr.	0.1857	8.0	15.0	0
*Cupania furfuracea* Radlk.	0.1776	26.0	35.0	1.25
*Astrocaryum aculeatissimum* (Schott) Burret	0.1618	20.0	30.0	1.00
*Vitex polygama* Cham.	0.1585	2.0	5.0	0
*Cecropia hololeuca* Miq.	0.1562	12.0	15.0	1.60
*Siparuna reginae* (Tul.) A.DC.	0.1552	14.0	25.0	0
*Phyllostemonodaphne geminiflora* Kosterm.	0.1484	4.0	10.0	1.67
*Calyptranthes lucida* Mart. ex DC.	0.1415	18.0	30.0	0
*Pseudopiptadenia contorta* (DC.) G.P. Lewis & M.P. Lima	0.1412	8.0	15.0	1.50
*Roupala sculpta* Sleumer	0.1336	10.0	15.0	1.20
*Ormosia* cf. *minor* Vogel	0.1332	8.0	20.0	1.00
*Marlierea obscura* O. Berg	0.1318	2.0	5.0	0
*Hirtella angustifolia* Schott ex Spreng.	0.1278	4.0	5.0	2.25
*Himatanthus bracteatus* (A. DC.) Woodson	0.1268	16.0	25.0	1.00
*Ocotea diospyrifolia* (Meisn.) Mez	0.1231	10.0	20.0	1.20
*Psychotria vellosiana* Benth.	0.1226	30.0	40.0	0
*Tibouchina arborea* Cogn.	0.1129	6.0	15.0	1.83
*Ecclinusa ramiflora* Mart.	0.1121	6.0	10.0	2.20
*Casearia arborea* (Rich.) Urb.	0.1116	32.0	50.0	0
*Licaria* sp.	0.1063	8.0	15.0	2.80
*Helicostylis tomentosa* (Poepp. & Endl.) Rusby	0.0989	20.0	40.0	0
*Guarea guidonia* (L.) Sleumer	0.0913	6.0	5.0	2.50
*Miconia lepidota* Schrank & Mart. ex DC.	0.0903	8.0	15.0	1.20
*Pera glabrata* (Schott) Poepp. ex Baill.	0.0878	6.0	15.0	0
*Protium heptaphyllum* (Aubl.) Marchand	0.0860	12.0	25.0	0
*Jacaranda puberula* Cham.	0.0839	14.0	25.0	1.33
*Miconia* sp.	0.0794	18.0	20.0	1.20
*Stryphnodendron polyphyllum* Mart.	0.0792	8.0	20.0	2.00
*Copaifera langsdorffii* Desf.	0.0758	2.0	5.0	1.67
*Ocotea glaziovii* Mez	0.0731	4.0	10.0	1.17
*Ocotea* sp.1	0.0673	8.0	20.0	1.20
*Psychotria carthagenensis* Jacq.	0.0628	8.0	20.0	0
*Trichilia martiana* C. DC.	0.0577	2.0	5.0	0
*Leretia cordata* Vell.	0.0576	4.0	10.0	0
*Erythroxylum citrifolium* A. St.-Hil.	0.0557	14.0	25.0	0
*Ocotea daphnifolia* (Meisn.) Mez	0.0540	4.0	5.0	0
*Cybistax antisyphilitica* (Mart.) Mart.	0.0534	4.0	5.0	0
*Ocotea laxa* (Nees) Mez	0.0473	6.0	5.0	0
*Mollinedia oligantha* Perkins	0.0447	4.0	10.0	0
*Strycnos* sp.	0.0422	2.0	5.0	1.00
*Polyandrococos caudescens* (Mart.) Barb. Rodr.	0.0367	2.0	5.0	1.00
*Ocotea brachybotrya* (Meisn.) Mez	0.0365	2.0	5.0	0
*Pera heteranthera* (Schrank) I.M. Johnst.	0.0342	10.0	20.0	0
*Copaifera lucens* Dwyer	0.0337	2.0	5.0	1.33
*Mabea fistulifera* Mart.	0.0302	4.0	10.0	0
*Maytenus samydaeformis* Reissek	0.0300	8.0	20.0	0
*Leretia cordata* Vell.	0.0299	4.0	10.0	0
*Persea* sp.	0.0294	2.0	5.0	0
*Licania octandra* (Hoffmanns. ex Roem. & Schult.) Kuntze	0.0276	6.0	15.0	1.20
*Urbanodendron* cf. *bahiense* (Meisn.) Rohwer	0.0275	4.0	10.0	0
*Erythroxylum cuspidifolium* Mart.	0.0270	8.0	15.0	0
*Eugenia* sp.2	0.0268	2.0	5.0	0
Malpighiaceae 2	0.0265	2.0	5.0	0
*Solanum inaequale* Vell.	0.0253	2.0	5.0	0
*Kielmeyera excelsa* Cambess.	0.0241	4.0	10.0	0
*Eugenia speciosa* Cambess.	0.0226	4.0	10.0	0
*Siparuna guianensis* Aubl.	0.0215	6.0	15.0	1.20
*Swartzia oblata* R.S.Cowan	0.0212	2.0	5.0	0
*Lecythis lanceolata* Poir.	0.0201	2.0	5.0	0
*Alchornea sidifolia* Müll. Arg.	0.0200	4.0	5.0	0
*Senegalia* sp.	0.0199	4.0	10.0	0
*Calyptranthes brasiliensis* Spreng.	0.0198	4.0	5.0	1.17
*Euterpe edulis* Mart.	0.0190	6.0	15.0	1.33
*Pseudobombax grandiflorum* (Cav.) A. Robyns	0.0175	2.0	5.0	0
*Calophyllum brasiliense* Cambess.	0.0158	2.0	5.0	2.40
*Lecythis lanceolata* Poir.	0.0156	4.0	10.0	1.20
*Myrsine coriacea* (Sw.) R. Br. ex Roem. & Schult.	0.0153	2.0	5.0	1.00
*Guarea kunthiana* A. Juss.	0.0151	4.0	5.0	2.00
*Myrcia splendens* (Sw.) DC.	0.0148	2.0	5.0	0
*Eugenia* sp.3	0.0143	2.0	5.0	0
*Eugenia* sp.1	0.0140	2.0	5.0	1.50
*Cupania* sp.	0.0134	2.0	5.0	0
*Handroanthus heptaphyllus* Mattos	0.0132	4.0	10.0	0
*Calyptranthes* cf. *lanceolata* O. Berg	0.0129	2.0	5.0	0
*Ocotea* sp.2	0.0125	2.0	5.0	0
*Licania octandra* (Hoffmanns. ex Roem. & Schult.) Kuntze	0.0120	2.0	5.0	0
*Trichilia casaretti* C. DC.	0.0116	2.0	5.0	0
*Pouteria bangii* (Rusby) T.D. Penn.	0.0113	4.0	5.0	1.20
*Cupania schizoneura* Radlk.	0.0108	2.0	5.0	1.20
*Guarea macrophylla* Vahl	0.0107	4.0	10.0	2.67
*Inga tenuis* (Vell.) Mart.	0.0106	2.0	5.0	1.00
*Miconia prasina* (Sw.) DC.	0.0101	4.0	10.0	0
*Pourouma guianensis* Aubl.	0.0096	4.0	10.0	0
*Tabebuia* sp.3	0.0089	2.0	5.0	0
*Eugenia magnifica* Spring	0.0088	2.0	5.0	0
*Myrcia* sp.1	0.0086	2.0	5.0	0
*Calyptranthes* sp.	0.0070	2.0	5.0	0
*Machaerium brasiliense* Vogel	0.0069	2.0	5.0	0
*Duguetia pohliana* Mart.	0.0064	2.0	5.0	0
*Tachigalli pilgeriana* (Harms) Oliveira-Filho	0.0064	2.0	5.0	0
*Cupania oblongifolia* Mart.	0.0061	2.0	5.0	1.33
*Brosimum guianense* (Aubl.) Huber	0.0056	2.0	5.0	1.25
*Gomidesia* sp.	0.0052	2.0	5.0	0
*Cyathea corcovadensis* (Raddi) Domin	0.0049	2.0	5.0	0
*Eugenia tinguyensis* Cambess.	0.0049	2.0	5.0	0
Myrtaceae 1	0.0046	2.0	5.0	0
*Couepia venosa* Prance	0.0044	2.0	5.0	0
*Simaba* sp.	0.0042	2.0	5.0	0

UV = use-value, Ab.Freq= absolute frequency, Ab.Dens.= absolute density.

The state of forest provides much information about past economic activities. For instance in spite of the impacts produced by lumber industry, it is, the principal reason for the existing traditional knowledge even four decades after that activity had ceased. There is an intricate web of relationships in natural areas that keep in contact people and situations like: rural workers; lumber industry activities; scientists; environmentalists; NGOs; local politicians; forest resources scarcity; trustworthy economic alternatives to rural workers; and conflicting interests among groups.

Studies in Amazonian forest areas have shown the highest use-values, from 60 up to 98% of species ([Piñedo-Vásquez et al. 1990], [Phillips et al. 1994], [Galeano 2000], [Soler-Alarcón and Peixoto 2008]). The only available data for the Atlantic Rain Forest is 100% ([Cunha and Albuquerque 2006]), where all of 42 shrub and tree species were used by local human communities.

A total of 30 uses were registered for the species that were grouped in eight categories, and among them, construction (11 indications), and technology (10) comprised most of the indications. Most of the species demonstrated low levels of use-versatility, ranging from one up to four uses.

The traditional specialists studied recognized the importance of conserving the remnant forests as a way of protecting the resources they use, thus presenting the possibility of recruiting them as front-line agents for the conservation and management of the Atlantic Forest, a forest that is currently too reduced of its original area in Rio of Janeiro State.

[Cunha and Albuquerque (2006]) reported finding six up to ten uses for 15 species (among the 42 reported as being useful), while [Soler-Alarcón and Peixoto (2008]) reported that 43% of the 185 species inventoried in an area of Amazonian Forest, ranging from six up to ten uses.

**Figure 1:**
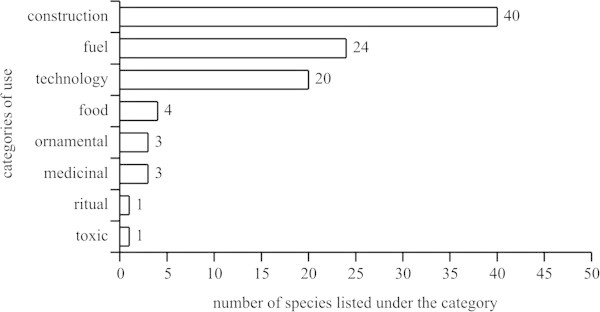
**Distribution of the numbers of species in use-categories mentioned by traditional specialist on ethnobotanical survey in rural community, municipality of Silva Jardim, RJ, Brazil.**

The construction category had the largest number of useful species cited (40 spp.), followed by firewood (24), and then technology (20) (Figure 1). When considering the total numbers of citations, construction had 51.77% of the use-indications of the local specialists, while 22.79% were indications for technological purposes, and 18.56% for firewood.

The background of the traditional specialists had a very strong influence on the results of the research reported here, as these men were predominantly ex-wood harvesters and most of their knowledge about the forest was focused on tree species used as timber, as could be observed during the fieldwork. There was an intense activity in traditional economy as a consequence of the lumber industry in the 1940’s that were spread throughout the central coastal area of Rio of Janeiro. Their former occupations explain their accuracy in recognizing fully 78.20% of the arboreal individuals sampled and 53.49% of the species encountered.

**Figure 2:**
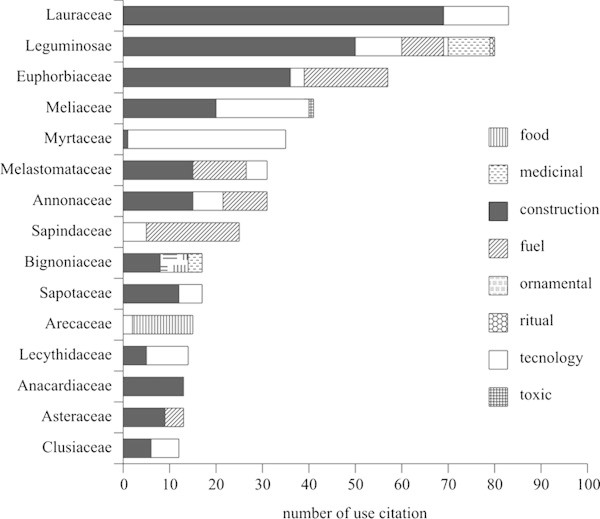
**Distribution of the numbers of citations by use-categories of plants mentioned by traditional specialist an ethnobotanical survey in a rural community, municipality of Silva Jardim, RJ, Brazil.**

Most of the species had low UV and only a few had a high number of uses according to the traditional specialists, indicating that each species has essentially only one typical use. Considering the distribution of the number of use-citations per family in the various use-categories (Figure 2), Lauraceae stands out in terms of the absolute number of citations, although it demonstrates low versatility among the different use categories, with its component taxa being restricted to the categories of construction and technology. Fabaceae, with a slightly smaller number of citations than Lauraceae, is distributed among six distinct use-categories: medicinal, construction, firewood, ornamental, ritualistic, and technological. The third family in number of citations was Euphorbiaceae, being cited in three categories (construction, firewood, and technology).

[Cunha and Albuquerque (2006]) noted the importance of Fabaceae, Melastomataceae, Burseraceae, and Anacardiaceae in numbers of uses, the majority of which were related to construction and technology. In studies undertaken in two indigenous areas in the Amazonian Forest in Brazil, [Prance et al. (1987]) indicated the families Arecaceae, Apocynaceae, Clusiaceae, and Chrysobalanaceae as being most important for the Ka’apor Amerindians; while for the Tembé indigenous tribe Arecaceae, Chrysobalanaceae, Annonaceae, and Lauraceae stood out. It is worth pointing out that the families Fabaceae, Lauraceae, Melastomataceae, and Arecaceae are widely distributed in the country, occurring in many different vegetation formations, and have significant roles in the characterization of those physiognomies.

The selective extraction of lumber for construction constitutes the most common use of forest products, and they are used in residences and in shelters for domestic animals. Among all of the species encountered, 58% were used for this, and 70% of those species where used exclusively in this category. The families most indicated for construction use are Lauraceae, Fabaceae, Euphorbiaceae, Meliaceae, Melastomataceae, Annonaceae, Anacardiaceae, and Sapotaceae.

Firewood is the second largest use and included 24 species. The families most used are Sapindaceae and Euphorbiaceae, the latter having the greatest number of use-citations. Wood is directly used for cooking and for preparing manioc flour, as well as to produce charcoal. Some residences have their own charcoal-manufacturing kilns made of mud and called a charcoal hearth.

In the technology category, wood is used basically for making handles for tools as well as crates for transporting farm produce. According to some of the traditional specialists, the practice of making these wooden crates greatly reduced the number of certain plant species in the region due to excessive demand in the past for box-wood to supply an industry installed in the region.

The food uses of forest species were concentrated in Arecaceae - the source of palm-hearts. Within the fragment studied, three palm tree species are harvested for this purpose: *Astrocaryum aculeatissimum*, *Euterpe edulis*, and *Polyandrococos caudescens*. The search for *Euterpe edulis*, the most appreciated palm-heart, was responsible for the accentuated destruction of the forests of Rio of Janeiro, as this species was once very abundant along the Atlantic coast, growing even on the mountainsides. Vestiges of cut palms were found in the sample plots.

Only three species were recognized as having medicinal properties: *Jacaranda puberula* was indicated for treating itching, using the leaf to prepare an infusion. *Copaifera langsdorffii* and *C. lucens* were indicated for treating afflictions of the digestive system, using the plant sap. The ornamental category had three species: *Jacaranda micrantha*, *J. puberula*, and *Tibouchina arborea*. The traditional specialists indicated that these plants were considered ornamental due to their large size and showy flowers. Two categories had only a single species: *Guarea guidonia* is considered toxic to cattle; and the seeds of *Ormosia* cf. *minor* are used for adornment in Afro-Brazilian cult rituals.

The data demonstrated that the species in the fragment with high UV appeared to have low relative density (RD) – suggesting that the species with the greatest versatility had reduced populations – this association was not in fact found to be significant (UV = 1.59 – 2.57*RD; r^2^ = 0.004; F = 0.266; *p* > 0.05). Of the three species that stood out as having the highest UV: *Xylopia sericea*, *Lecythis lanceolata*, and *Guarea macrophylla*; the last two demonstrated very low RD in the study fragment (0.54 and 0.27 respectively).

A similar situation was noted in the association between high UV and high relative dominance (RDo). The results also were not significant (UV = 1.45 + 8.64*RDo; r^2^ = 0.049; F = 3.447; *p* > 0.05), although the species with the greatest UV was the species with the greatest RDo (4.54) - which may be related to the size of the population (17 individuals) and to their bulk.

In this forest fragment, at the species level, the use-value was not dependent on either the species abundance or their dominance. It may be an indicative for conservation, because the low density or even rarity must not necessarily prevent a plant species to be taken for human use, what may endanger them. Some plant species of rare occurrence in Atlantic Rain Forest inventories, with a single individual per hectare, may represent more than 50% of the sampled taxa, which contributes to the high diversity indexes of these forests ([Guedes-Bruni et al. 2009]). This rarity pattern must be taken into account before drawing conclusions about the eventual results from the management by local people.

**Figure 3:**
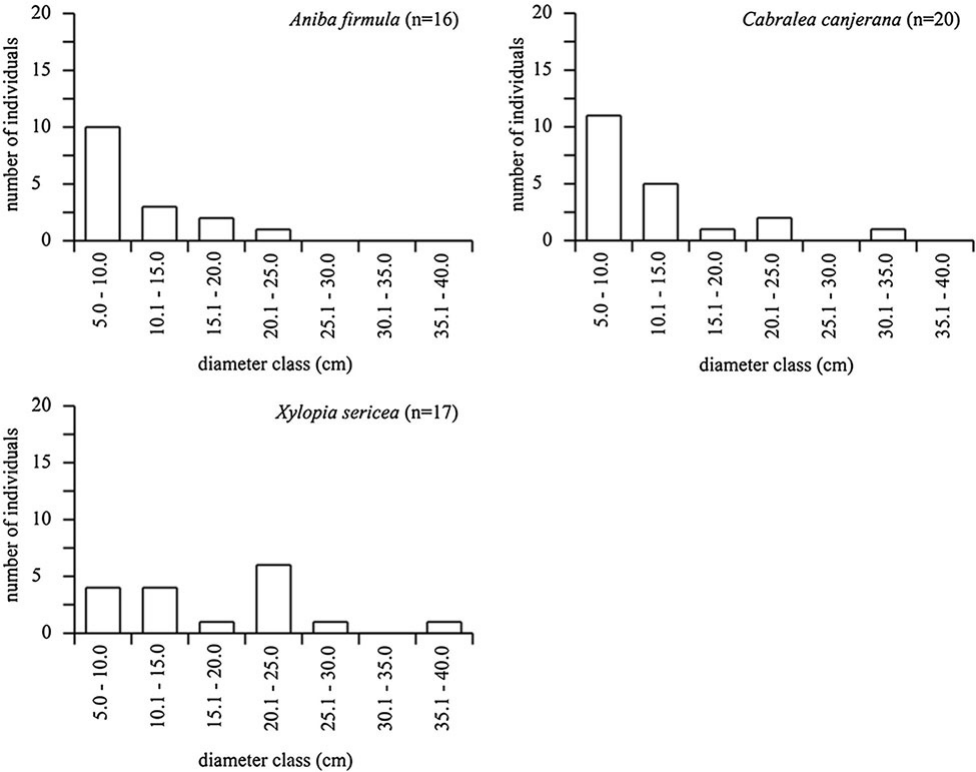
**Diameter distribution of the three tree species with the greatest UV and largest numbers of individuals in an ethnobotanical survey in rural community, municipality of Silva Jardim, RJ, Brazil.**

The analysis of diameter distributions of the three species with the highest UV and populations of greater than 15 individuals (Figure 3) demonstrated that *Aniba firmula* and *Cabralea canjerana* had inverted “J” distribution patterns, indicating typical demographic regeneration patterns. *Xylopia sericea*, on the other hand, did not demonstrate this pattern, suggesting problems during its regeneration process in a given stage of the community, or an alteration directly related to the taxon. In examining the structural descriptors of this species and associating them to ethnobotanical data, it could be seen that *Xylopia sericea* is heavily used for constructing roofs for local residences, and trees with stem diameters between 5 and 20 cm are preferentially harvested. This specific diameter selection results in reduced numbers of individuals in the first two diameter classes in the forest fragment studied. Those individuals not selectively harvested will grow and occupy the canopy layer at maturity, making up the tallest individuals in the fragment.

**Figure 4:**
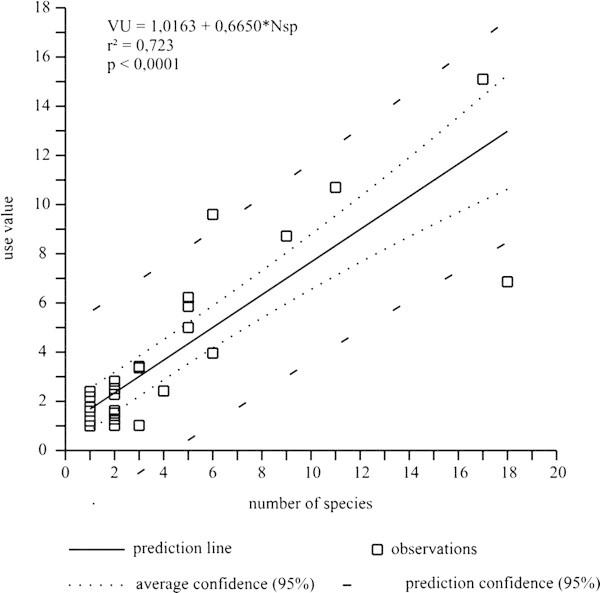
**Linear regression analysis of the use-values of the plant families and their respective use-values from an ethnobotanical survey in rural community, municipality of Silva Jardim, RJ, Brazil.**

The association between the use-value of a given family and its richness (Figure 4) was significant and positive (UV_f_ = 1.02 + 0.66*N.Spp; r^2^ = 0.723; *F* = 70.542; *p* < 0.0001). The best predictor of a species’ usefulness is therefore associated with family richness, corroborating the results reported by ([Moerman 1996]).

**Figure 5:**
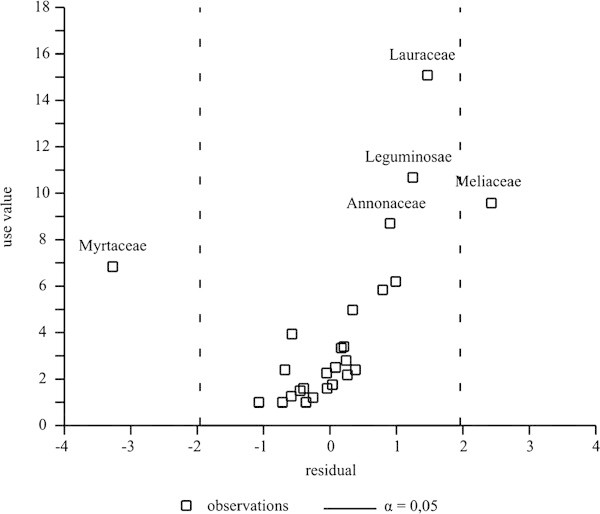
**Analysis of the residual standard errors obtained by the linear regression between the use-values of the families and their respective use-values from an ethnobotanical survey in rural community, municipality of Silva Jardim, RJ, Brazil.**

In the residual plots (Figure 5), the families are represented by observation points. Assuming α = 0.05, the family Myrtaceae is apparently under-used in light of its high species richness, considering the low use-values attributed to it by the traditional specialists. In spite of the fact that this family is recognized as having a large number of species in the Atlantic Forest, these plants were basically only used to make tool handles, as they have smooth trunks and their soft-textured wood is comfortable to grip. Additionally, the species sampled possess a shrubby habitus and thus less valued by the traditional specialists who are more interested in lumber species. Meliaceae, however, was over-used due to its low richness but high use-value. Six species of this family occurred in the area, all of them valued for producing wood used in house construction and for making furniture.

Larrère *et al* (2003) observed that “*Biodiversity is the resultof the interaction ofnatural processes and humanactivities acting over longtime scales. Human activitiesare not necessarily unfavorableto biological diversity. Conservation,in itself, is notrestricted solely to naturebut includes human culture*”. As such, traditional knowledge concerning the uses and populations sizes of forest species should be consulted when preparing proposals for management activities that intend to conserve plant resources and when developing sustainable alternatives to guarantee the permanence and well-being of those rural communities.

Interdisciplinary investigations that evaluate the structure of forest fragments together with the use of those resources by traditional rural communities can provide important information for future in situ conservation programs, while at the same time offering the prospect of sustainable management and use of natural products by those same communities.

## Conclusions

The knowledge accumulated by local specialists during their work harvesting native trees had influenced their manner of seeing the forest, and even though they were assisting a scientific research project (and were aware of the importance of conserving the forest as a whole), these specialists consistently attributed greater value to arboreal species, especially those valued as lumber species. When considering the total number of use-citations to construction, technological, and firewood uses, the association between past experience as lumber extractors and theirs actual activities as rural workers is expressed.

The association between the importance of these resources and their structural descriptors were not observed. The plant resource selection is related to specific demand, regulated by different activities of the subsistence, as cooking, making handles for tools, building shelter for animals and home construction. Conservation actions to some species should be case to case, considering the species density, geographic occurrence, reproduction, and importance as a plant resource.

The way of use and the demand on the forest resource by traditional people should be taken into consideration in conservation programs, considering the immediate dependence of rural workers on forest resources to benefit their agricultural products (e.g. mandioc flour), as well as for their constructions. A survey about forest plant species conservation should be individually made for each useful species, also considering their distribution patterns in nature.

## Authors’ information

Ariane Luna Peixoto^d^

^d^CNPq fellow
